# About the management of patients with wandering spleen in pregnancy and postpartum

**DOI:** 10.1007/s00404-025-08009-3

**Published:** 2025-06-24

**Authors:** Brunialti Daniela, Prader Sonia

**Affiliations:** Department of Gynecology and Obstetrics, Bressanone, Alto Adige Italy

**Keywords:** Wandering spleen, Torsion, Pregnancy, Postpartum

## Abstract

**Purpose:**

This study aimed to review the literature with regard to the management and outcome in pregnant and postpartum females with wandering spleen.

**Methods:**

The literature was reviewed for articles regarding the following search terms: ‘pregnancy’, ‘postpartum’, ‘torsion’ and ‘wandering spleen’.

**Results:**

17 articles were found in medical literature from 1907 to 2022. Case reports were divided up into 2 groups: antepartum group that counts 12 articles and the postpartum group with 5 articles. The median age of these females was 28 years. All patients had symptoms across both groups: abdominal pain (n=13), vomiting (n=5), thrombocytopenia (n=4), nausea (n=3) and thrombocytosis (n=2). 16 patients underwent splenectomy and 1 pregnant woman received conservative management during pregnancy. 15 patients had no post-operative complications. 1 woman had an incomplete abortion in the 1 st trimester and 1 female a stillbirth in the 3 rd trimester.

**Conclusion:**

Wandering spleen in pregnancy and in puerperium is a rare condition with many different possible manifestations. Up to now literature has favored laparoscopic or open splenectomy as treatment for it. From asymptomatic patients to urgent and emergent cases, the diagnosis of a wandering spleen must be included when a pregnant or postpartum woman complains about an abdominal palpable mass and recurrent abdominal pain.

## Introduction

Wandering spleen, also called ectopic or dislocated spleen, is a rare clinical picture and an unusual entity [1]. Wandering spleen is characterized by a distinct mobility and consequently alteration of spleen’s usual position [2]. This clinical picture has an incidence rate of under 0.2% [3]. Until 2012 medical literature indicates almost 500 cases of WS [3], diagnosed in patients ranging from 3 months to 82 years of age [4]. The presentation of a WS is often seen in females between their second-to-fourth decades of life [5]. It is supposed that women in reproductive age and especially hormonal variation during pregnancy may be the impact factor for ligamentous strengthening and this clinical picture in pregnant and postpartum females [5]. Far less is known about the wandering spleen in pregnant females. Medical literature has reported only a few case reports about WS in pregnancy and puerperium. Some case reports were described as emergencies and others as quite simple surgical interventions [6–9]. Nearly all medical reports in pregnancy or puerperium are describing splenectomy as treatment for a wandering spleen [6–9], but splenectomy is a powerful therapeutic procedure, and the risks should be weighed against the potential benefits in each individual case [10], especially in pregnant females. This review takes a critical look at the management of a wandering spleen in pregnant and postpartum patients referring to complications that this clinical picture can bring.

## Materials and methods

The literature was reviewed for articles regarding following search terms: ‘pregnancy’, ‘postpartum’, ‘torsion’ and ‘wandering spleen’. Because of the scarce incidence of this clinical picture and in this context poor number of articles found in Medline database, we decided to include all case reports that were published up to now in medical journals like: “Bulletin of the National Research Centre”, “Open Journal of Obstetrics and Gynecology”, “International Journal of Clinical Research”, “The Journal of Medical Research”, “Ultrasound in Obstetrics and Gynecology”, “International Journal of Tropical Medicine”, “Nepalese Journal of Radiology” and “Clinical and Experimental Obstetrics & Gynecology”. In total we found 20 case reports about the WS during pregnancy and in the postpartum period. The inclusion criteria were articles written in English about pregnant or postpartum females. 17 articles met these inclusion criteria. Case reports were divided up into 2 subgroups: in the antepartum and in the postpartum period. The timeframe in the antepartum group was set from the sixth week of pregnancy until childbirth, whereas the timeline in the postpartum period was determined from first day to 6 week postpartum. The primary endpoints of this analysis were the type of surgical treatment and complications by managing this clinical picture. Data were collected in following areas: (a) type of surgery, (b) patient’s age at surgery, (c) number of previous pregnancies, (d) initiated symptoms in week of pregnancy, (e) imaging diagnosis, (f) postoperative complications and (g) immunological handling (Table 1).

**Table 1:** Procedures for WS in pregnancy and puerperium

First author, year of publication	Procedure
H. Meek, 1907	Open splenectomy
D. Lincolin Lewis, 1962	Open splenectomy
Cobellis et al., 2001	Conservative treatment
Gilman et al., 2002	Open splenectomy
Parvaiz et al., 2004	Open splenectomy
Huang et al., 2006	Open splenectomy
Akinola et al., 2009	Open splenectomy
Ghazeeri et al., 2010	Open splenectomy
Yúcel et al., 2010	Laparoscopic splenectomy
Lahiri et al., 2010	Open splenectomy
Abbey et al., 2013	Open splenectomy
Anyfantakis et al., 2013	Laparoscopic splenectomy
Mamadou et al., 2018	Open splenectomy
Terro et al., 2020	Laparoscopic splenectomy
Han et al., 2020	Open splenectomy
Gupta et al., 2021	Open splenectomy
Crepaz et al., 2022	Laparoscopic splenectomy

## Results

20 articles were found in medical literature from 1907 to 2022; 17 articles met the inclusion criteria as delineated above. The antepartum group counts 12 articles, the postpartum group counts 5 articles.

Regarding to the surgical procedure, 16 patients underwent splenectomy and 1 pregnant woman received conservative management during pregnancy. In the antepartum group 8 women got an open laparotomy, whereas 3 patients had a laparoscopic splenectomy. From the postpartum group 4 patients had an open splenectomy and 1 patient a laparoscopic surgery. In the antepartum group splenectomies were performed earliest at sixth week till 36th week of pregnancy. In the postpartum group patients got splenectomy earliest immediately after childbirth till 1-month post-partum.

The age of these patients across both groups ranges from 18 to 40 years; the median age of these women is 28.

In the antepartum group eight women were primigravida; three females were multigravida. In the postpartum group, one patient was a primipara and other three females were multipara.

All females across both groups showed symptoms, associated with the ectopic spleen. In the antepartum group, ten patients had abdominal pain, five patients vomiting and one patient nausea. Four patients had thrombocytopenia. In the post-partum group, three women had abdominal pain, two nausea and two patients’ thrombocytosis. In patients from the antepartum group symptoms associated with the WS began in a timeframe starting at the sixth week of pregnancy till 32nd week of pregnancy. In patients from the postpartum group symptoms associated with the WS initiated immediately postpartum till 1 month post-delivery.

Imaging diagnosis in the antepartum group was in 8 patients the ultrasound; additional MRI was used in 2 patients. In one woman was used X-ray for detection of the WS and in another pregnant female culdocentesis. Imaging diagnosis in the postpartum group was CT-scan in 4 patients and in 1 woman ultrasound and X-ray.

Surgical outcome after splenectomy was uneventful in 15 patients. 2 patients from the antepartum group were hemodynamically unstable: in one patient was executed open laparotomy in the third month of pregnancy; 3 day post-surgery the patient had an incomplete abortion. In the other woman was performed open splenectomy in the 28th week of pregnancy; on the third postoperative day she expelled a stillbirth.

Regarding to the immunological handling 8 patients across both groups received vaccination against Streptococcus pneumoniae, Hemophilus influenzae and Neisseria meningitidis. In the antepartum group 2 patients got vaccination post splenectomy, 1 patient received vaccination before splenectomy and 2 patients received vaccination post splenectomy and post childbirth. One of these patients got additional antibiotic chemoprophylaxis after splenectomy. In the postpartum group 3 patients got vaccination; 1 patient got vaccination before splenectomy and 2 of them had vaccination post-surgery and got additional antibiotic chemoprophylaxis as prevention (Tables 2 and 3).

**Table 2:** Clinical findings in the antepartum group

First author	Age	Previous pregnancies	Symptoms	Diagnostic imaging	Complications	Surgical outcome
H. Meek	32	no data	abdominal pain, constipation	none	splenic torsion	uneventful
D. Lincolin Lewis	28	2nd	backache, nausea, vomiting, abdominal pain	X-ray	spleen’s torsion, adherences to the small intestine	uneventful
Cobellis	40	1st	abdominal pain	ultrasound	-	-
Pavaiz	27	1st	backache, right shoulder tip pain, dizziness, syncope, thrombocytopenia, fetal bradycardia, vaginal bleeding	none	shock, lacerated and partially infarcted spleen	uneventful
Akinola	29	1st	dizziness, vomiting, weakness, abdominal pain, abdominal swelling	ultrasound	shock, rotated spleen, bleeding from splenic vein	stillbirth
Ghazzeri	23	1st	vomiting, thrombocytopenia	ultrasound; MRI	twisted splenic pedicle, splenic infarction	uneventful
Yúcel	24	1st	abdominal distension, constipation	ultrasound; MRI	-	uneventful
Lahiri	18	1st	abdominal pain and distension	culdo-centesis	shock, rotation of the spleen, multiple ruptures on the surface	incomplete abortion
Mamadou	26	3rd	abdominal and pelvic pain with vomiting	ultrasound	twisted spleen	unevenful
Terro	24	2nd	abdominal pain, thrombocytopenia	CT-scan, ultrasound	partially torted splenic pedicle with microinfarcts	uneventful
Gupta	22	1st	abdominal pain, bilious vomiting	ultrasound	partially infarcted spleen	uneventful
Crepaz	31	1st	abdominal pain, thrombocytopenia	ultrasound	acute splenic infarction	uneventful

**Table 3:** Clinical findings in the postpartum group

First author	Patient’s age	Nr. of pregnancies	Symptoms	Diagnostic imaging	Complications	Surgical outcome
Gilman	24	Multipara	Lower back pain, abdominal pain, nausea	CT-scan; MRI	Infarcted spleen, pancreatitis	Uneventful
Huang	35	7th	Thrombozytosis, leukocytosis	Ultrasound, abdominal X-ray	Splenic pedicle strangulation with thrombosis, ischemic spleen, distal pancreatic necrosis	Uneventful
Abbey	19	1st	Abdominal pain, thrombocytopenia	CT-scan	Torsion and ischemia of the spleen, thrombosis of splenic vein	Uneventful
Anyfantakis	34	No data	Epigastric pain, nausea, vomiting	CT-scan	Splenic torsion, signs of ischemia, thrombosis of splenic artery	Uneventful
Han	35	7th	Thrombozytosis	CT-scan	Splenic torsion	Uneventful

## Discussion

Historically, the first WS with torsion of the splenic pedicle during pregnancy was described in 1907 [11]. This case report described the first open splenectomy during pregnancy [11]. Under physiological conditions the spleen is attached on the left hypochondrium through the splenic pedicle that is formed by the gastrosplenic and splenorenal ligaments. The splenic pedicle includes the splenic artery, vein and the tail of the pancreas [5, 12]. The result of elongated or absent suspensory splenic ligaments is a long splenic mesentery and hypermobility of the spleen [5, 12]. As etiological factors congenital and acquired influences are discussed [13]. If fusion of the dorsal mesogastrium with the dorsal abdominal wall during the second month of embryogenesis fails, it is evaluated as congenital anomaly [13], whereas pregnancy, trauma and splenomegaly may count as acquired risk factors [5].

Until 2012 medical literature indicates almost 500 cases of WS across all population groups [4]; these do not account for asymptomatic cases. Therefore, the prevalence of this condition might be higher. Until now only 20 case reports of WS in pregnancy and puerperium have been documented in medical literature [7, 9]. Therefore, the knowledge about WS in pregnancy is scarce. 2 case reports were published in the twentieth century, whereas 15 articles were written in the twenty-first century. Regarding to future, medical articles about WS in pregnant and puerperal women will increase if we consider ongoing availability of imaging diagnosis in pregnancy in various countries.

Nowadays, laparoscopic splenectomy seems to be the best choice for removal of the spleen for benign hematologic disease [8]. Laparoscopy allows for a thorough inspection of the intraperitoneal cavity, finding and repositioning the spleen in its anatomical position without a major abdominal incision [8]. The first report of a laparoscopic splenectomy for WS in pregnancy was published 12 years ago [8]. Since the publication from Yücel et al. only 2 other case reports with laparoscopic splenectomy during pregnancy were published [14, 15]. In these case reports laparoscopic splenectomy seems to be a feasible and a safe procedure in pregnant patients [8, 14, 15]. However, a laparoscopic approach in pregnant females depends on availability and expertise of the surgeon [16]. During surgery continuous monitoring of fetal vitality is required [8]. As mentioned above open laparotomy for splenectomy seems to be the choice to be for hemodynamically unstable patients [7, 19, 20]. In medical literature no case report about splenopexy during pregnancy and in puerperium is available. Because of high recurrence after splenopexy and the use of vaccines that lowers the risk of overwhelming post splenectomy sepsis, most surgeons have chosen splenectomy as treatment for a WS [8].

In our analysis we could not sign a direct connection between the number of previous pregnancies of the patient and the incidence of the WS. In the antepartum group 8 women were primigravida and 3 patients were multigravida. In the postpartum group 3 patients were multipara; 2 of them have had 7 previous pregnancies. By the lack of case reports it is difficult to make a significant statement for it.

The clinical presentation in females during pregnancy and puerperium ranges from an asymptomatic abdominal mass to more commonly acute intermittent or chronic abdominal pain. Splenic torsion is a rare complication of a WS and is a rare cause of acute abdominal pain in pregnancy or puerperium [9]. It is a rare condition characterized by increased splenic mobility due to the absence or laxity of its suspensory ligaments [17]. Especially, in pregnancy where diminished peritoneal cavity volume as a result of the gravid uterus and a maximal third trimester increase in whole blood volume might be the predisposing factors in pregnancy for splenic torsion [7]. In the acute presentation, a dislocated and torted spleen during pregnancy poses a serious threat to life due to thrombosis, bleeding or infarction of the spleen. In this case splenectomy is the procedure to choose [18]. Despite splenectomy that was performed in two hemodynamically unstable pregnant females, stillbirth and incomplete abortion followed some days after surgery [19, 20]. This underscores the potential damage that a WS in pregnancy can bring. In pregnant females, especially giving a vaginal birth, another possible complication of WS is the risk of the splenic rupture. Therefore, in medical literature no case report describes vaginal childbirth under the condition of a WS.

If we consider that surgical outcome in all patients, that were treated for WS was uneventful, we could assume that splenectomy in open laparotomy and in laparoscopy seem to be quite tolerable interventions even if the patients are pregnant, but it is true that by the lack of case reports for this clinical picture it is difficult to make a powerful statement. Maybe in future when case reports will increase, we could make a more precise statement.

From the immunological point of view we cannot make any reliable statements, because case reports describe just the immediate surgical outcome after splenectomy and no long-term course. Common complications can be: haemorrhage, thromboembolic disorders, subphrenic abscess, chest infection and overwhelming post-splenectomy infection. Overwhelming bacterial sepsis as a complication in patients post splenectomy seems now infrequent because of vaccinations, prophylactic penicillin, and prompt medical attention at the first sign of fever [21, 22]. In these case reports no infections were described after splenectomy during hospitalization. Other complications of asplenia are still investigated like a higher risk of pulmonary hypertension [10] or other platelet disorders [10]. Nowadays, it is recommended to make vaccination against streptococcus pneumoniae, hemophilus influenzae and neisseria meningitidis before splenectomy.

## Conclusion

Wandering spleen in pregnancy and in puerperium is a rare condition with many different possible manifestations. From asymptomatic patients to urgent and emergent cases, the diagnosis of a wandering spleen must be included in the differential diagnosis, when a pregnant woman complains about an abdominal palpable mass and recurrent abdominal pain. Literature has favored laparoscopic or open splenectomy as treatment for it. Considering that no splenopexy was carried out we cannot recommend a spleen-preserving trail in pregnant patients with a wandering spleen. Laparoscopic or open splenectomy is feasible with no reported complications. For better knowledge wandering spleen in pregnant and postpartum females we are looking forward to more reports in medical literature. More information about the long term post-surgical course in patients that underwent splenectomy during pregnancy is needed for decision support. Splenic torsion, though rare should be considered a differential diagnosis in pregnant and postpartum patients, who present with symptoms of shock and acute abdomen.

## Abbreviation

WS: Wandering spleen

## References

[1] Maingot R (1980) Splenectomy: indications and technique; Abdominal operation, 7^th^ ed., vol 2. Appleton-Century-Crofts, New York, pp 679–708

[2] Buehner M, Baker MS (1992) The wandering spleen. Surg Gynecol Obstet 175(4):373–387 (**PMID: 1411897**) 1411897

[3] Puranik AK, Mehra R, Chauhan S, Pandey R (2017) Wandering spleen: a surgical enigma. Gastroenterol Rep (Oxf) 5(3):241–243. 10.1093/gastro/gov034. (**Epub 2015 Aug 3. PMID: 26240117; PMCID: PMC5554386**) 26240117 10.1093/gastro/gov034 PMC5554386

[4] Lane TM, South LM (1999) Management of a wandering spleen. J R Soc Med 92(2):84–85. 10.1177/014107689909200211. (**PMID:10450220;PMCID:PMC1297068**) 10450220 10.1177/014107689909200211 PMC1297068

[5] Qazi SA, Mirza SM, Muhammad AM, Al Arrawi MH, Al-Suhaibani YA (2004) Wandering spleen. Saudi J Gastroenterol 10(1):1–7 (**PMID: 19861821**) 19861821

[6] Sentürk M, Kasikci YS (2022) Wandering spleen, which is torsioned with the distal pancreas. Ulus Travma Acil Cerrahi Derg 28(9):1363–1365. 10.14744/tjtes.2021.34288. (**PMID:36043920;PMCID:PMC10315951**) 36043920 10.14744/tjtes.2021.34288 PMC10315951

[7] Parvaiz A, Chandran S, Karim A, Kumar K, Jeffrey P, Lagattolla NR (2004) Torted and ruptured wandering spleen presenting as a surgical emergency in pregnancy. ScientificWorldJournal 6(4):1035–1037. 10.1100/tsw.2004.208. (**PMID:15632981;PMCID:PMC5956381**) 10.1100/tsw.2004.208 PMC5956381 15632981

[8] Yücel E, Kurt Y, Ozdemir Y, Gun I, Yildiz M (2012) Laparoscopic splenectomy for the treatment of wandering spleen in a pregnant woman: a case report. Surg Laparosc Endosc Percutan Tech 22(2):e102–e104. 10.1097/SLE.0b013e318246beb5. (**PMID: 22487633**) 22487633 10.1097/SLE.0b013e318246beb5

[9] Gilman RS, Thomas RL (2003) Wandering spleen presenting as acute pancreatitis in pregnancy. Obstet Gynecol 101(5 Pt 2):1100–1102. 10.1016/s0029-7844(02)02617-0. (**PMID: 12738115**) 12738115 10.1016/s0029-7844(02)02617-0

[10] Weledji EP (2014) Benefits and risks of splenectomy. Int J Surg 12(2):113–119. 10.1016/j.ijsu.2013.11.017. (**Epub 2013 Dec 3 PMID: 24316283**) 24316283 10.1016/j.ijsu.2013.11.017

[11] Meek H (1907) Dislocated spleen with torsion of pedicle complicating pregnancy: removal of spleen: delivery at term. Br Med J 1(2416):928. 10.1136/bmj.1.2416.928. (**PMID:20763184;PMCID:PMC2357262**) 20763184 10.1136/bmj.1.2416.928 PMC2357262

[12] Lewis GA, Byrne MP (1981) Wandering spleen. Am Surg 47(6):275–277 (**PMID: 7247110**) 7247110

[13] Lam Y, Yuen KK, Chong LC (2012) Acute torsion of a wandering spleen. Hong Kong Med J 18(2):160–162 (**PMID: 22477742**) 22477742

[14] Crepaz L et al (2022) The wandering spleen: case report of laparoscopic splenectomy in a pregnant woman. Bull Natl Res Centre 46:273

[15] Terro J, El-Chamaa B, Abdallah S, Jammoul K, El Lakkis R, El-Helou E, Al-Raishouni MA, Shibli A, Al-Shami J, Abtar HK (2020) A case report: wandering spleen in a young primiparous woman treated by laparoscopic splenectomy. Int J Clin Res 1(1):29–33

[16] Mamadou T, Bernadin K, Ibrahim A, Michelle M, Estelle D, Ismaêl L, Bernadette N, Serges E, Amos K, Junior Z, Roger L, Bamourou D (2018) Acute volvulus of a wandering spleen on pregnancy: a case report. Open J Obstet Gynecol 8:300–305. 10.4236/ojog.2018.84032

[17] Alimoglu O, Sahin M, Akdag M (2004) Torsion of a wandering spleen presenting with acute abdomen: a case report. Acta Chir Belg 104(2):221–223 15154585 10.1080/00015458.2004.11679541

[18] Benevento A, Boni L, Dionigi G, Ferrari A, Dionigi R (2002) Emergency laparoscopic splenectomy for “wandering” (pelvic) spleen: case report and review of the literature on laparoscopic approach to splenic disease. Surg Endosc 16(9):1364–1365 12045854 10.1007/s00464-002-4213-6

[19] Akinola O, Fabamwo A, Rabiu K, Tayo A, Omodele F, Akinola R (2009) Splenic torsion in pregnancy: a case report. Int J Trop Med 4:129–131

[20] Lahiri S, Dasgupta N, Mondal AU (2010) A case of splenic torsion and rupture presenting as ruptured ectopic pregnancy. J Surg Case Rep 2010(10):4. 10.1093/jscr/2010.10.4. (**PMID:24945841;PMCID:PMC3649179**) 24945841 10.1093/jscr/2010.10.4 PMC3649179

[21] Sarangi J, Coleby M, Trivella M, Reilly S (1997) Prevention of post splenectomy sepsis: a population based approach. J Public Health Med 19(2):208–212 9243438 10.1093/oxfordjournals.pubmed.a024611

[22] Zarachi MH, Rosner F (1986) Rarity of failure of penicillin prophylaxis to prevent splenectomy sepsis. Arch Intern Med 140:1207–1208 3521520

